# Classification of partial seizures based on functional connectivity: A MEG study with support vector machine

**DOI:** 10.3389/fninf.2022.934480

**Published:** 2022-08-18

**Authors:** Yingwei Wang, Zhongjie Li, Yujin Zhang, Yingming Long, Xinyan Xie, Ting Wu

**Affiliations:** ^1^Department of Radiology, Affiliated Hospital of Nanjing University of Chinese Medicine, Nanjing, China; ^2^College of Intelligence and Computing, Tianjin Key Laboratory of Cognitive Computing and Application, Tianjin University, Tianjin, China; ^3^National Laboratory of Pattern Recognition, Institute of Automation, Chinese Academy of Sciences, Beijing, China; ^4^Brainnetome Center, Institute of Automation, Chinese Academy of Sciences, Beijing, China; ^5^Department of Magnetoencephalography, Nanjing Brain Hospital, Affiliated to Nanjing Medical University, Nanjing, China

**Keywords:** temporal lobe epilepsy, resting-state functional connectivity, MEG, machine learning, classification

## Abstract

Temporal lobe epilepsy (TLE) is a chronic neurological disorder that is divided into two subtypes, complex partial seizures (CPS) and simple partial seizures (SPS), based on clinical phenotypes. Revealing differences among the functional networks of different types of TLE can lead to a better understanding of the symbology of epilepsy. Whereas Although most studies had focused on differences between epileptic patients and healthy controls, the neural mechanisms behind the differences in clinical representations of CPS and SPS were unclear. In the context of the era of precision, medicine makes precise classification of CPS and SPS, which is crucial. To address the above issues, we aimed to investigate the functional network differences between CPS and SPS by constructing support vector machine (SVM) models. They mainly include magnetoencephalography (MEG) data acquisition and processing, construction of functional connectivity matrix of the brain network, and the use of SVM to identify differences in the resting state functional connectivity (RSFC). The obtained results showed that classification was effective and accuracy could be up to 82.69% (training) and 81.37% (test). The differences in functional connectivity between CPS and SPS were smaller in temporal and insula. The differences between the two groups were concentrated in the parietal, occipital, frontal, and limbic systems. Loss of consciousness and behavioral disturbances in patients with CPS might be caused by abnormal functional connectivity in extratemporal regions produced by post-epileptic discharges. This study not only contributed to the understanding of the cognitive-behavioral comorbidity of epilepsy but also improved the accuracy of epilepsy classification.

## Introduction

According to the World Health Organization, about 50 million people worldwide suffered from epilepsy (Fiest et al., 2017). In recent years, epilepsy has been proposed to be a disorder of the brain network caused by hypersynchrony of neuronal activity (Zhang et al., 2011; Richardson, 2012). Epilepsy could be divided into focal, generalized onset, and unknown onset based on clinical manifestations and electroencephalogram (EEG) in line with the new International League Against Epilepsy ILAE criteria in 2017 (Scheffer et al., 2017). Depending on whether awareness was impaired, focal seizures were classified as aware or impaired awareness seizures, which are also known as “simple partial seizure (SPS)” and “complex partial seizure (CPS),” respectively (Falco-Walter et al., 2018).

Temporal lobe epilepsy (TLE), a common neurological disorder originates in the temporal lobe (de Lanerolle et al., 1989). Seizures types in TLE primarily incorporated SPS, which can cause focal motor or selective emotional or visual changes with relatively preserved consciousness, and CPS, which has more sophisticated clinical manifestations, including epigastric paresthesia, cognitive impairment, paresthesia, and automatisms (Proposal for Revised Clinical and Electroencephalographic Classification of Epileptic Proposal for Revised Clinical Electroencephalographic Classification of Epileptic Seizures., 1981; Depaulis et al., 1997). The EEG is mostly normal in SPS, and abnormal EEG changes were transient and were generally restricted peaks or paroxysmal activity in the temporal lobe regions (Inoue et al., 2000; Janszky et al., 2004).

During the past few decades, increasing evidence have linked epileptic cognitive impairment and loss of consciousness to diffuse brain network changes (Blume, 2002; Blumenfeld et al., 2009; Xu et al., 2009; Englot et al., 2010; González et al., 2019; Hermann et al., 2021). The duration of CPS invariably exceeded 30 s, and the discharge position was deeper. Meanwhile, CPS tended to spread to the brainstem or contralateral hemisphere, resulting in extensive neurological alterations (Stayman and Abou-Khalil, 2011; Hauf et al., 2013). “Network inhibition hypothesis” was a new theory of CPS proposed by Blumenfeld (Blumenfeld et al., 2004, 2009; Guye et al., 2006), was According to the theory, focal discharges in the temporal lobe interfered with brainstem–diencephalon arousal system, and then inhibited ascending reticular activation system, which indirectly brought forth impaired cortical function and loss of consciousness (Steriade, 1970; Motelow et al., 2015). Pathophysiological studies had shown a significant increase in slow-wave activity in the frontoparietal neocortex and an enhanced rate of diffusion of fast EEG activity from the medial temporal lobe to the contralateral side during CPS compared to SPS (Englot et al., 2017). In animal studies, it was also found that blood oxygenation level-dependent in the hippocampus of TLE increased, while it decreased in the cortex and thalamus (Motelow et al., 2015). This suggested that cortical and subcortical structures are involved in regulating consciousness. Studying alterations in resting-state brain functional connectivity could be conducive to explore the mechanisms underlying cognitive dysfunction in individuals with epilepsy. Memory deficiencies in individuals with CPS were associated with compensatory increases in the hippocampus. There was also evidence that executive dysfunction was relevant to a reduced resting-state functional connectivity in the frontoparietal lobe (Park et al., 2017; Ives-Deliperi and Butler, 2021; Li et al., 2022). Language dysfunction was associated with reduced functional connectivity in the frontotemporal lobe language network. EEG researches have revealed that even if the bilateral pikes in patients with SPS and CPS originated in disparate brain regions, the discharges spread to the same area, the temporal lobe base (Sirven et al., 1996). This indicated that SPS and CPS may have shared network nodes in the temporal lobe. However, the mechanisms by which CPS brain network alterations are associated with impaired consciousness and behavioral abnormalities have not been systematically studied.

Unlike other diseases, the first symptom defined the epilepsy type, consciousness turned into a “watershed,” differentiated SPS from CPS. Nevertheless, a flat dichotomy may result in neglecting the other clinical symptoms (Muayqil et al., 2018). Currently, there is a lack of direct studies on brain network alters among SPS and CPS individuals. Therefore, our study hypothesized that aberrant differences in functional connectivity of CPS and SPS were vital nodes involved in the regulation of brain network consciousness and behaviors. Furthermore, seizures were primarily self-reported by the patient, and even with EEG testing, the underreporting rate was still as high as 50% (Glauser et al., 2010; Elger and Hoppe, 2018; Verdru and Van Paesschen, 2020). Especially, when epilepsy originates in deep or non-dominant regions, the initial weak signal may not be obtained on scalp EEG (Benbadis et al., 2020). Compared to EEG, the sensitivity and specificity of magnetoencephalography (MEG) for localization of minute EEG activity were higher than that of EEG (van Mierlo et al., 2014). In contrast to MRI, even though functional brain networks were widely used in MRI (Salma et al., 2019; McKavanagh et al., 2021), the time span for information processing was only milliseconds to seconds when the brain was in a resting state. Therefore, the selection of MEG with higher localization accuracy for the analysis of functional connectivity differences was more promising for detecting subtle changes in the brain (Kakisaka et al., 2013; Nissen et al., 2018; van Klink et al., 2019).

Prior to this study, we preprocessed MEG data from patients with SPS and CPS and normal controls, and performed a source level analysis. The results of the coherence analysis showed that the functional connectivity of patients with both SPS and CPS was lower than that of normal individuals in the whole brain, while there was no significant difference between the two groups. Therefore, to address the problems of low efficiency and easy misdiagnosis in manual identification of EEG signals, we chose machine learning to further differentiate the differences between patients with SPS and CPS (Fallahi et al., 2021). At present, machine learning has shown sound application prospects for various neurological diseases incorporating epilepsy (Craley et al., 2020; Gleichgerrcht et al., 2020; Pavel et al., 2020). (Li et al., 2018; Bharath et al., 2019). In a previous study, SVM was able to distinguish temporal lobe epilepsy from benign epilepsy in healthy controls or central temporal spikes (Jin and Chung, 2017; Sriraam and Raghu, 2017; Yang et al., 2020). In addition, the use of SVM could also distinguish between resected and unresected regions based on preoperative interictal MEG data in epileptic patients. Therefore, the present study intends to use SVM combined with two sample selection approaches to explore the differences in functional connectivity between the two groups at rest.

## Materials and methods

### Patient population

This study was part of a research program on neuroimaging in epilepsy. It consecutively recruited 40 patients with TLE between 2015 and 2020. Inclusion criteria were as follows: 1. in line with the International League Against Epilepsy (ILAE, 1989) diagnostic criteria for epilepsy; 2. partial epilepsy diagnosed by V-EEG observation and medical history; 3. the clinical and EEG characteristics were onefold SPS or CPS; 4. individuals had no visible lesions in structural MRI images.; 5. able to cooperate with the inspection and the head movement during MEG examination was no more than 5 mm. Exclusion criteria: 1. combined with a history of generalized seizure ; 2. suffered from other types of paroxysmal illness (e.g., mental illness, severe systemic illness, etc.); 3. implants that may seriously interfere with MEG and MRI data collection (such as dentures, cochlear implants, pacemakers, etc.); 4. unable to cooperate with MEG and MRI examination. Of the 40 patients we recruited, All of them underwent MEG and MRI scans at Nanjing Brain Hospital affiliated with Nanjing Medical University. All individuals have read and signed voluntary and written informed consent for the study prior to enrollment, according to the standards set by the ethical committee of Nanjing Brain Hospital of Nanjing Medical University, which approved this study.

### MEG resting state acquisition

All studies were performed in a magnetically shielded room by using our 275-channel whole-head biomagnetometer (VSM MedTech, Coquitlam, BC, Canada). The full head sensor of the 275 super-conducting quantum interference device (SQUID) was used to measure the brain magnetic field in the direction perpendicular to the scalp. Three electrically active coils were placed as fiducial markers at the nasion and 1 cm anterior to the left and right tragus to measure the position of each person's head relative to the MEG sensor.

All individuals did not have seizures at least 16 h before the examination. If the frequency of seizures was less than once a week, individuals were required to stop anti-epileptic drugs for 2 days and overnight sleep deprivation (increased seizure activity). MEG recording is performed at a sample rate of 1,200 Hz for twenty 120-s recordings with the patient in the state of rest and their eyes closed to detect interictal MEG sharp waves and spikes, as well as bursts of rhythmic activity.

### Anatomical MRI

MRI data were collected using a US GE Signa NV/i 1.5 T super-conducting magnetic resonance apparatus, and the head of the subject was fixed with a sponge pad. The routine anatomical MRI data were acquired to detect structural details. T1-weighted image scans were obtained, with following parameters: TR/TE = 1,750 ms/24 ms, FA = 90°, matrix = 256 × 256, FOV = 24 × 24 mm2, slice thickness = 6 mm, slice gap = 2 mm, and acquired slices = 16. Coronal T2-FLAIR-weighted image scans were also obtained, with following parameters: TR/TE = 8,400 ms/135 ms, FA = 150°, matrix = 256 × 256, FOV = 24 × 24 mm^2^, slice thickness = 16 mm, slice gap = 2 mm and acquired slices = 16.

### Data preparation

All MEG recordings were reviewed by two experienced epileptologists at Nanjing Medical University, and the peaks of all epileptic spikes and inter epileptic discharge were marked manually based on the MEG recordings.

#### Artifact rejection and subtraction of inter-spikes relative-stable activity

All analyses were done with custom-written MATLAB (Mathworks, Natick, MA) scripts and FieldTrip (http://www.ru.nl/fcdonders/fieldtrip/). Firstly, all spikes and abnormal discharge before and after the 10 s time period were excluded. Then we short-cut the remaining segments with a non-overlapped length of 10 s and further extracted the sub-segments which are free from jump-like artifacts and muscular artifacts. To ensure the same length of the remaining sub-segments, we further removed the ones with a length <4 s and short-cut the others with a length of 4 s. The number of segments per subject varied from 33 to 169 at last. The total number of segments for all the individuals with CPS was 1,306, and the total number of segments for all the individuals with SPS was 1,526.

#### Filtering and removing artifact

The remaining signals were low-pass-filtered at 70 Hz and high-pass-filtered at 1 Hz with notch-filtered around 50 Hz (the vertical refresh rate of the LCD projector). Furthermore, to increase the speed of the following data analysis, the data were down-sampled to 100 Hz. In addition, we removed blink artifacts and ECG noise from data by using Independent Component Analysis (ICA).

### Source reconstruction

We performed source reconstruction after preprocessing using a partial cannonical correlation/coherence (PCC) (Mukuta and Harada, 2014). Specifically, first, the subject-specific T1-weighted MR images were re-sliced and segmented to obtain brain/skull boundary by using FieldTrip. Then, we generated the individual cortical meshes with > 1,00,000 vertices per hemisphere by using the Freesurfer package (version 5.3.0) (surfer.nmr.mgh.harvard.edu), and we downsampled them to 8,196 nodes in all by using MNE Suite (martinos.org/mne/stable/index.html). Next, the downsampled cortical sheet was coregistered to the sensor-based coordinate system with FieldTrip. Finally, the volume conduction for the forward model was computed by using FieldTrip's “single shell” method, and the source reconstruction was computed between 1 and 40 Hz with a partial canonical correlation/coherence (PCC) method.

### Functional connectivity analysis

For each segment time course, we calculated functional connectivity (FC) by the imaginary part of the coherency index between the time courses at every two sheets after source reconstruction. To reduce the dimensionality of the FC matrix, we applied a parcellation scheme according to the Destrieux Atlas, which consists of 74 parcels in the whole cortical surface (Destrieux et al., 2010). Therefore, each FC matrix was merged to a size of 74-by-74.

### Classification of FC matrix between CPS and SPS

**Figure 1:**
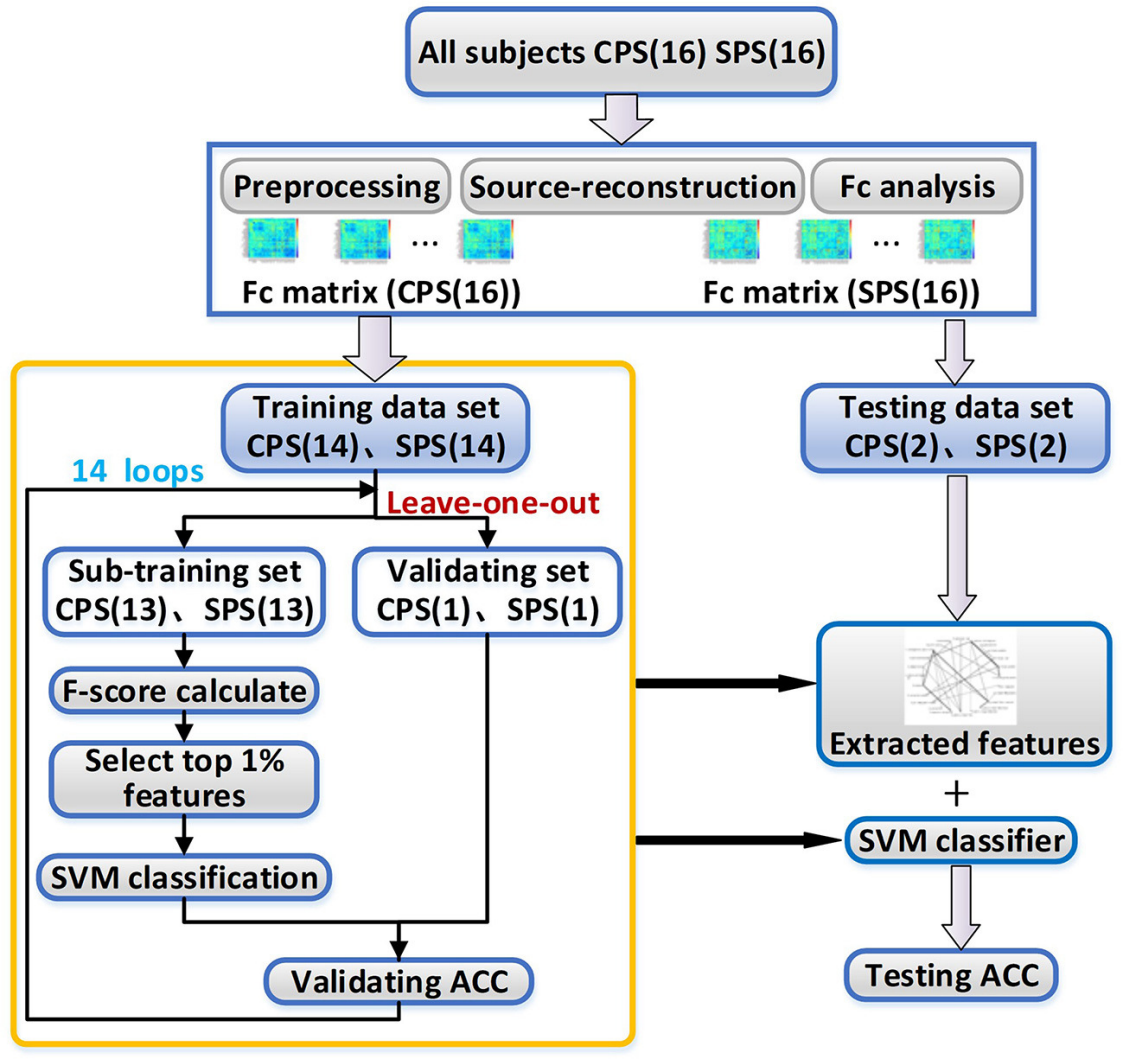
The working flowchart of the proposed framework. **CPS**, complex partial seizures; **SPS**, simple partial seizures; **SVM**, support vector machine; **ACC**, the accuracy rate.

In the next step, we tried to find the functional connectivity pairs that were different between CPS and SPS groups by using a nonlinear classification method with feature extraction and leave-one-out loop. The features which contributed well and were stable for separating the two patient groups were considered to indicate the corresponding cortical pairs having obvious distinct functional connectivity between them. The operational flowchart of the proposed framework is shown in Figure 1.

Specifically, as shown in Table 1, we divided all the subjects into 16 subsets, and each subset had one patient with CPS and one with SPS. For each subset, the number of CPS FC matrices was basically equal to the number of SPS FC matrices. We randomly selected two subsets as the testing set, and the others were considered as a training set for classification.

**Table 1:** Specific information of the 14 subsets of subjects.

	**Training set**														**Testing set**	
**Subset**	**1**	**2**	**3**	**4**	**5**	**6**	**7**	**8**	**9**	**10**	**11**	**12**	**13**	**14**	
CPS	Su12 (102)	Su01 (73)	Su03 (78)	Su05 (81)	Su17 (33)	Su10 (34)	Su18 (37)	Su09 (40)	Su07 (72)	Su14 (84)	Su08 (117)	Su04 (125)	Su16 (144)	Su15 (169)	Su02 (75)	Su04 (42)
SPS	Su11 (105)	Su16 (77)	Su32 (123)	Su33 (146)	Su14 (51)	Su22 (57)	Su09 (58)	Su18 (59)	Su15 (64)	Su12 (89)	Su07 (117)	Su23 (148)	Su03 (150)	Su06 (168)	Su24 (34)	Su31 (80)

The numbers in bracket indicating the number of FC matrices belonging to the subject. SPS, simple partial seizures. CPS, complex partial seizures.

To improve the generalization ability of the classification model as possible as we can, we adopted a leave-one-out method for the training processing in this work. For each loop, we selected 13 subsets from the training set as sub-training set and left one subset as validating set to select the best hyper-parameters for the classification model. For the sub-training set, we selected the FC features, which have stable and great contribution for separating the CPS and SPS, as the methods introduced in “Feature extraction” section. Then, we trained SVM classifier as introduction in “Support vector machine classifier”. And then, we repeated this process 14 times for all possible selection options.

After leave-one-out training, we counted the overlap rate of all appeared features among 14 times leave-one-out training loop and extracted the features with the highest (≥12/14) overlap rate as a reliable feature assemblage. Finally, we extracted the corresponding feature values in the testing dataset as the input for the classifier and performed the classification as we trained. The test accuracy rate here was calculated. The method for feature extraction and classification were introduced below in detail.

#### Feature extraction

To reduce the duplicate information in the FC matrix which was symmetrical, we extracted its lower triangular part and its main diagonal as the vector. Each value in the vector is a feature for classification, indicating an FC strength between a pair of cortical areas. The total number of features contained in each vector is C${}_{\text{\_}74^{\wedge }2+74=}$2,775. It is important to select the most essential features using feature selection before entering the classifier (Guyon and Elissee, 2003) when the number of features is much more than the number of subjects used in classification.

To more effectively enhance the efficiency of computation and avoid the high-dimensional and small-sample-size problem for classification, we further measured the classification capability of each feature by the F-score method (Chen et al., 2006) and removed the features which were irrelevant or has low correlation to the classification (Guyon and Elissee, 2003). F-score is a simple and generally quite an effective technique that can measure the ability of a feature to discriminate between two classes of samples. Given a set of training samples *x*_*k*_, *k = 1*, ⋯, *m*, then the F-score of the ith feature is defined as follows:


(1) $$F_{i}=\frac{{\left({\bar{x}}_{i}^{\left(-\right)}-{\bar{x}}_{i}\right)}^{2}+{\left({\bar{x}}_{i}^{\left(+\right)}-{\bar{x}}_{i}\right)}^{2}}{\frac{1}{n_{+}-1}\sum_{k=1}^{n_{+}}{\left(x_{k,i}^{\left(+\right)}-{\bar{x}}_{i}^{\left(+\right)}\right)}^{2}+\frac{1}{n_{-}-1}\sum_{k=1}^{n_{-}}{\left(x_{k,i}^{\left(-\right)}-{\bar{x}}_{i}^{\left(-\right)}\right)}^{2}}$$

where ${\bar{x}}_{i}$ is the average of the *i*^th^ feature in the whole data set, whereas ${\bar{x}}_{i}^{\left(+\right)}$, ${\bar{x}}_{i}^{\left(-\right)}$are the average of the *i*^th^ feature in CPS, and SPS data sets, respectively; *n*_+_ and *n*_−_ represent the number of samples in CPS and SPS data sets, respectively; and $x_{k,i}^{\left(+\right)}$ and $x_{k,i}^{\left(-\right)}$ indicate the *i*^th^ feature of the *k*^th^ sample in the two groups' data sets, respectively. F-score is positive, and the larger the F-score of a feature is the stronger its ability to distinguish two categories of samples.

**Figure 2:**
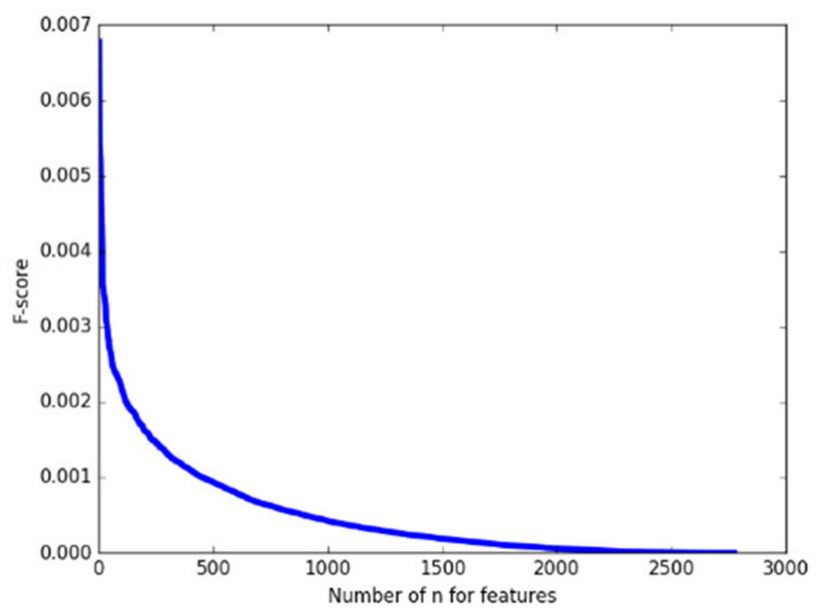
The F-score of 2,775 features.

As a pretest, we calculated the F-score of 2,775 features on the whole training set first and arranged them in descending order. As shown in Figure 2, it can be found that the F-score curve of 2,775 features declines rapidly and there are a large number of feature F-scores close to zero, suggesting only a very small number of features have a good contribution to classification while the others are redundant. Therefore, in the following classification processing, we extracted the features with the top 1% F–scores from the training subset as the input for classification.

#### Support vector machine classifier

The SVM is one type of binary supervised learning classifier that has been widely used in recent years (Ines et al., 2013) with a high ability for generalization (Vapnik, 2000). Rajpoot et al. (2015) used AP clustering combined with SVM to study functional connectivity changes in individuals with epilepsy and normal subjects. Its basic model is a linear classifier. For linearly separable data, SVM generates a separating hyperplane which separates the data with the largest margin. For linearly inseparable data, SVM can efficiently perform a non-linear one using kernel function Φ*(x)* (2), mapping their inputs into high-dimensional feature spaces.


(2) $$\begin{array}{r} \Phi \left(x\right):R^{n}\to R^{nh} \end{array}$$

By choosing a suitable Φ*(x)*, the SVM constructs an optimal separating hyperplane in higher-dimensional feature space to solve the linear-inseparable problem (Cantor-Rivera et al., 2015). The kernel function mentioned above may be any of the symmetric functions that satisfies the Mercel conditions (Dhanalakshmi et al., 2009). We selected the Radial Basis Function (RBF) in this work since it was the most frequently used kernel function, and there were two parameters *C* and γ, *which* must be preset before training the SVM classifier.

In the experiment, we implemented the scikit-learn, a machine learning tool package in python, to train the SVM classifier with parameter C of 1.2 and parameter γ of 5, and each sample was converted to scikit-learn input data format in this work.

## Results

Five patients had obvious head movement during scanning, defined as more than a 2 mm translation. Two patients refused to participate in further research and asked to withdraw. This meant that 32 patients met the stringent inclusion criteria and were finally included in the study. Among them, 16 patients (five male, 29.1 ± 8.63 years) were suffering from CPS and 16 patients (11 male, 23.5 ± 5.66 years) from SPS. The detailed demographic and electroclinical data are summarized in Table 2.

**Table 2:** Ictal semiology in the three groups of patients,the number of patients in each group having the concerning symptoms.

	**CPS (*n* = 16)**	**SPS (*n* = 16)**
Age (year)	29.11 ± 8.63	23.53 ± 5.66
Male (*n*, %)	5 (31.25)	8 (50)
Seizure duration (second)	104.06 ± 34.62	15.56 ± 6.12
Impairment of consciousness (*n*, %)	16 (100)	0 (0)
Oro-alimentary Automatisms (*n*, %)	13 (81.25)	5 (31.25)
Motor Automatisms (*n*, %)	15 (93.75)	2 (12.5)
Vegetative symptoms (*n*, %)	6 (37.5)	2 (12.5)

We calculated the classification accuracies from each validating subset in the leave-one-out loop to evaluate the effectiveness of our feature extraction method and SVM classifier. The results were summarized in Table 3. For all the 14 times of training, the validation accuracies varied from 72.39 to 88.73%, of which the mean is 79.87%.

**Table 3:** Validation accuracy for each leave-one-out training loop.

**# Subset for validation**	**1**	**2**	**3**	**4**	**5**	**6**	**7**	**8**	**9**	**10**	**11**	**12**	**13**	**14**
Acc(%)	88.73	76.55	81.28	74.44	85.06	81.02	83.58	76.32	88.29	80.56	75.13	77.85	72.39	76.98
Average	79.87%													

ACC, the accuracy rate.

**Figure 3:**
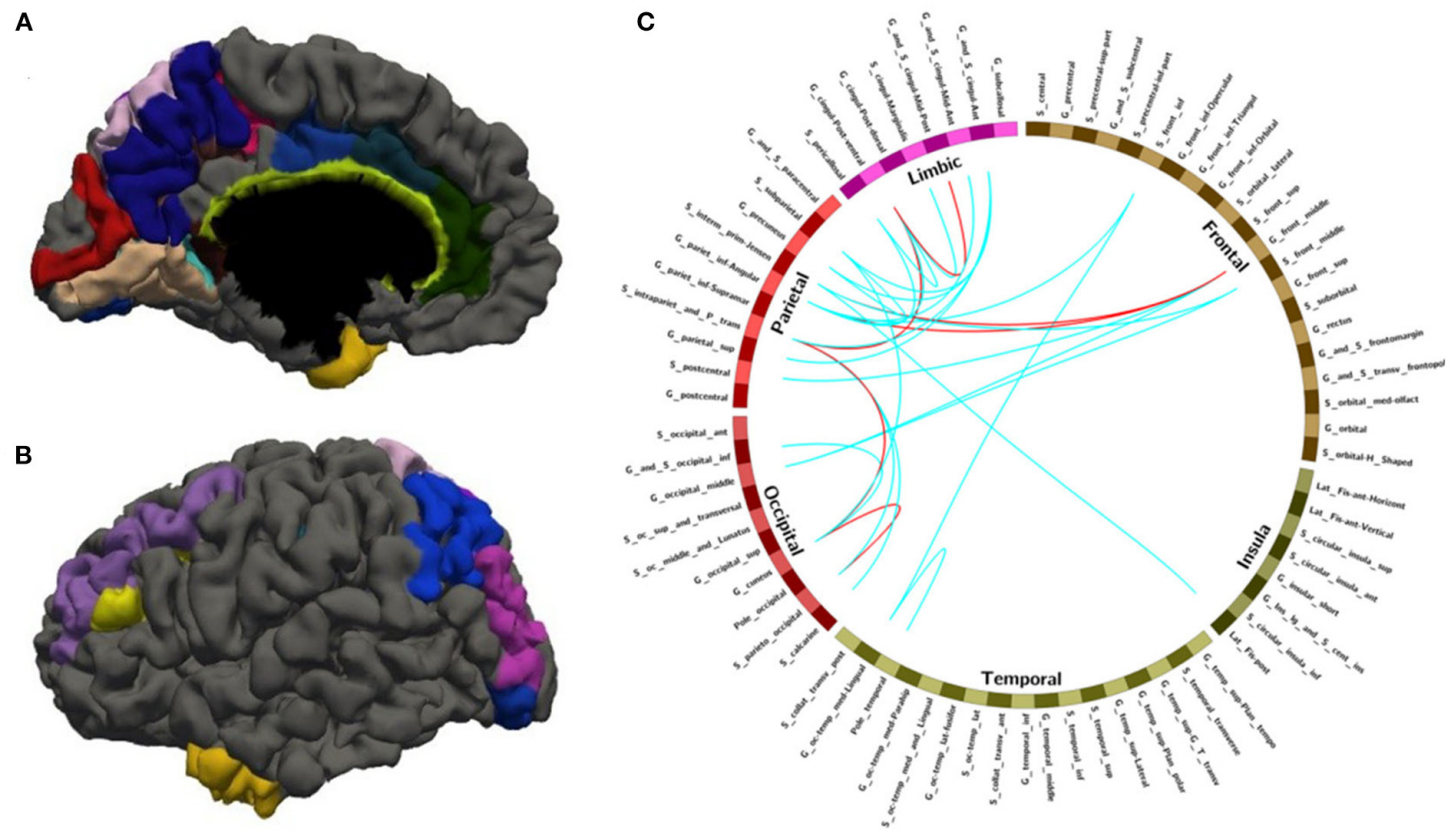
The nodes and edges involved in the FC features with high overlap rate (12/14) among training. **(A,B)** Regions involved in 28 FC features with overlapping ratios ≥12/14 across 14 training sessions. **(C)** Edges involved in 28 FC features with overlap ratio ≥12/14 in 14 training sessions.

After leave-one-out training, we got 28 FC features whose overlap rate was ≥12/14 among 14 times training. Figure 3 and Table 4 demonstrated the nodes and edges involved in these 28 FC features. According to the mean F-score value of each feature among 14 times training, we marked the FCs with great mean F-scores (higher than 75% of the highest mean F-score value among all the features) as a red line and colored the others in blue. They were mainly located within and between the parietal, the occipital, the frontal lobe, and the limbic system. For the temporal lobe and the insula, there was less different functional connectivity between SPS and CPS individuals. Specifically, as shown in Figure 3 and Table 4, the FCs with great mean F-scores were located between the middle frontal gyrus (G_front_middle), and the angular gyrus (G_pariet_inf-Angular), and Sulcus intermedius primus (of Jensen) (S_interm_prim-Jensen); between the Cuneus (G_cuneus) and the parieto-occipital sulcus (S_parieto_occipital), and the intraparietal sulcus (S_intrapariet_and_P_trans); and between the intraparietal sulcus (S_intrapariet_and_P_trans) and the posterior-ventral part of the cingulate gyrus (vPCC, G_cingul-Post-ventral); and between the posterior-ventral part of the cingulate gyrus (vPCC, G_cingul-Post-ventral) and the middle-posterior part of the cingulate gyrus and sulcus (pMCC, G_and_S_cingul-Mid-Post).

**Table 4:** The nodes and edges involved in the FC features with high overlap rate (12/14) among training.

**Index**	**ROI 1**	**ROI 2**
1	G_pariet_inf-Angular	G_front_middle
2	S_parieto_occipital	G_cuneus
3	S_interm_prim-Jensen	G_front_middle
4	S_intrapariet_and_P_trans	G_cingul-Post-ventral
5	S_intrapariet_and_P_trans	G_cuneus
6	G_cingul-Post-ventral	G_and_S_cingul-Mid-Post
7	S_pericallosal	G_pariet_inf-Angular
8	G_parietal_sup	G_and_S_cingul-Ant
9	S_front_inf	G_oc-temp_med-Lingual
10	G_occipital_middle	G_pariet_inf-Angular
11	S_intrapariet_and_P_trans	G_and_S_cingul-Ant
12	G_precuneus	G_and_S_cingul-Ant
13	G_pariet_inf-Angular	G_cingul-Post-ventral
14	S_interm_prim-Jensen	Lat_Fis-post
15	S_subparietal	G_precuneus
16	S_pericallosal	G_cingul-Post-ventral
17	S_subparietal	G_cuneus
18	G_pariet_inf-Angular	G_and_S_cingul-Mid-Ant
19	S_intrapariet_and_P_trans	S_calcarine
20	S_front_middle	G_occipital_middle
21	G_cuneus	G_and_S_occipital_inf
22	S_parieto_occipital	S_intrapariet_and_P_trans
23	S_subparietal	G_and_S_cingul-Ant
24	Pole_temporal	G_oc-temp_med-Lingual
25	S_postcentral	G_front_middle
26	S_cingul-Marginalis	G_cingul-Post-ventral
27	S_interm_prim-Jensen	S_front_middle
28	S_front_inf	G_pariet_inf-Angular

The pairs are ranked in descending order according to the mean F-score value of each feature among 14 times training.

According to these 28 FC features, we extracted the corresponding values in the testing dataset which contained 117 FC matrices for individuals with CPS and 114 FC matrices for individuals with SPS (as shown in the “Testing set” in Table 1). The test accuracy rate was 78.52%.

To further verify the stability of the results described above, we also adopted another criterion for sample selection in the training set. First, we mixed all the samples (FC matrices from all the time segments and all the subjects in the training set). For each time of the leave-one-out training procession, we randomly selected 90% of all the samples as a subset for training and the rest of 10% for validation. By calculating the F-score of each feature in the training subset, we took the features with top 1% F-score values as the input for the following SVM classifier. We also repeated this process 14 times. The validation accuracies in each leave-one-out loop are shown in Table 5, which varied from 78.55 to 87.21%, of which the mean is 82.69%. The final test accuracy rate reached upto 81.37%.

**Table 5:** Validation accuracy for each leave-one-out training loop with random sample selection.

**Group**	**1**	**2**	**3**	**4**	**5**	**6**	**7**	**8**	**9**	**10**	**11**	**12**	**13**	**14**
Acc(%)	83.25	81.94	79.27	78.55	87.21	84.35	81.13	84.57	86.88	82.46	86.12	81.97	82.48	84.59
Average	82.69%													

ACC, the accuracy rate.

**Figure 4:**
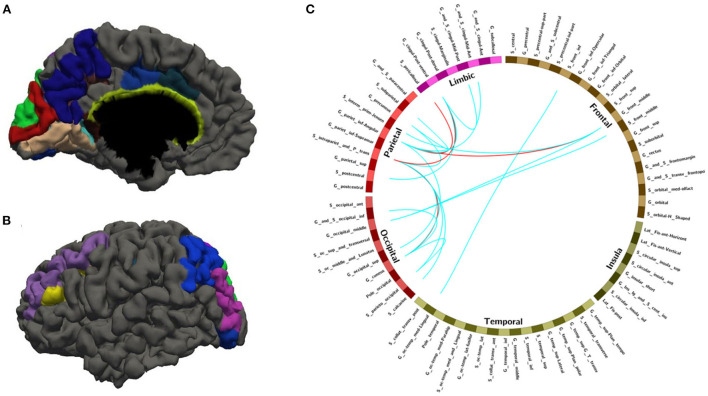
The nodes and edges involved in the FC features with a high overlap rate (12/14) among training with random sample selection. **(A,B)** Regions involved in 28 FC features with overlapping ratios ≥12/14 across 14 training sessions. **(C)** Edges involved in 28 FC features with overlap ratio ≥12/14 in 14 training sessions.

**Figure 5:**
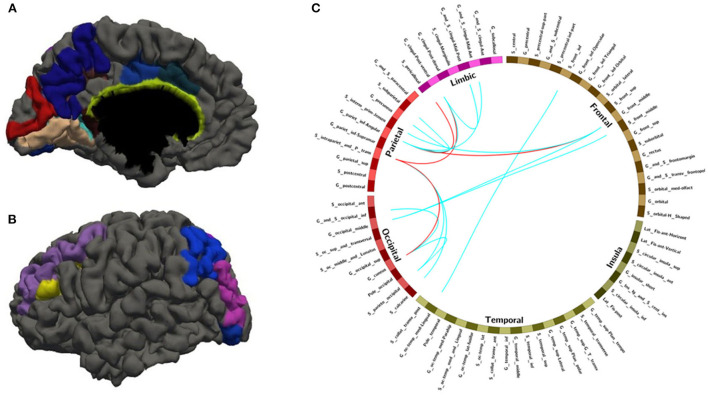
The overlapped ROIs and edges in both approaches **(A,B)** Regions involved in 28 FC features with overlapping ratios ≥12/14 across 14 training sessions. **(C)** Edges involved in 28 FC features with overlap ratio ≥12/14 in 14 training sessions. Red line: feature mean functional connections greater than the remaining 75% are marked with a red line. Blue line: no more than the remaining 75% of the feature mean functional connections are marked with blue lines.

With random sample selection in leave-one-out training loop, we got 20 FC features with an overlap rate ≥12/14. Figure 4 and Table 6 demonstrated their nodes and edges. They were mainly located within and between the parietal, the occipital, and the frontal lobe, and the limbic system, similar to the results in Figure 3. For the temporal lobe and the insula, there was less different functional connectivity between SPS and CPS individuals, either. In Figure 4, we marked the FCs with great mean F-scores (higher than 75% of the highest mean F-score value among all the features) as a red line, and colored the others in blue. Specifically, the FCs with great mean F-scores were located between the intraparietal sulcus (S_intrapariet_and_P_trans) and the posterior-ventral part of the cingulate gyrus (vPCC, G_cingul-Post-ventral) and the cuneus (G_cuneus), between the angular gyrus (G_pariet_inf-Angular) and the middle frontal gyrus (G_front_middle) and pericallosal sulcus (S_pericallosal). Comparing the results obtained with the two different ways of sample selection, we can find that there are many coincident ROIs and edges, as shown in Figure 5 and Table 6 (marked with ^*^).

**Table 6:** The nodes and edges involved in the FC features with high overlap rate (12/14) among training with random sample selection.

**Index**	**ROI 1**	**ROI 2**
1*	S_intrapariet_and_P_trans	G_cingul-Post-ventral
2*	S_intrapariet_and_P_trans	G_cuneus
3*	G_pariet_inf-Angular	G_front_middle
4*	S_pericallosal	G_pariet_inf-Angular
5*	S_parieto_occipital	S_intrapariet_and_P_trans
6*	S_front_inf	G_oc-temp_med-Lingual
7	S_intrapariet_and_P_trans	G_occipital_sup
8	G_pariet_inf-Angular	G_cuneus
9*	G_pariet_inf-Angular	G_cingul-Post-ventral
10*	G_occipital_middle	G_front_middle
11*	S_subparietal	G_precuneus
12*	G_cingul-Post-ventral	G_and_S_cingul-Mid-Post
13*	S_intrapariet_and_P_trans	S_calcarine
14*	S_front_middle	G_occipital_middle
15*	S_parieto_occipital	G_cuneus
16	S_intrapariet_and_P_trans	G_precuneus
17*	S_interm_prim-Jensen	G_front_middle
18	S_postcentral	G_cingul-Post-ventral
19*	G_cuneus	G_and_S_occipital_inf
20*	G_pariet_inf-Angular	G_and_S_cingul-Mid-Ant

The pairs are ranked in descending order according to the mean F-score value of each feature among 14 times training (^*^ after index represents the pair is overlapped in the results with two different ways of sample selection).

## Discussion

### Summary

In our pre-experiment, we found a significant decrease in functional connectivity in both groups of patients compared to healthy controls, which was consistent with previous studies (Ives-Deliperi and Butler, 2021). To date, few studies have focused on the resting state of SPS and CPS, especially the analysis of differences in brain functional connectivity between the two groups. We evaluated subjects' FC for exploring network connection markers for TLE with altered consciousness status. The synchronicity of activities between network nodes was calculated at the brain network level. A higher mean F-score value indicated a stronger activity of network nodes and a greater connectivity with other nodes. The network connection differences of CPS from SPS were distinguished by SVM. In this way, dissimilar regions in the functional connectivity of global networks in SPS and CPS were determined. The following primary results were made: (1) The SVM classification model used the optimal feature set of 28 functional connections calculated from MEG data to distinguish the CPS subjects from SPS at a mean accuracy of 81.37% (sensitivity = 81.1%; specificity = 81.54%) on test data. (2) Compared with SPS, individuals with CPS revealed a hyper-connectivity in several primary regions including intraparietal sulcus, transverse parietal sulcus of brissaud, middle frontal gyrus, callosal suleus, ventral posterior cingulate gyrus, cuneus, and inferior parietal marginal angular gyrus. By comparing the differences in FC between SPS and CPS, it was possible to explore the pathological basis of consciousness impairment and cognitive abnormalities.

### Relationship between CPS network connections and consciousness

There was no significant relationship between the occurrence of impaired consciousness in CPS and the functional connectivity of the epileptic region of origin (Najm, 2018). We did find that CPS and SPS functional connectivity differences were concentrated in extratemporal lobe regions. It was mainly distributed between the parietal, occipital, frontal. and limbic systems. This implied that the occurrence of CPS was not only associated with structural damages in the temporal lobe but also with abnormal brain network connectivity in extratemporal brain regions (Englot et al., 2010). Abnormal functional connectivity in these brain regions might accelerate the outward diffusion of temporal lobe discharge (Yoo et al., 2014; Sirin et al., 2020). This may lead to individuals who showed up with the loss of consciousness (Li et al., 2020).

Our observations were consistent with the “network inhibition hypothesis.” If seizures spread beyond the epileptogenic zone, consciousness may be vulnerable to impairment Bancaud et al., 1994; So, 1995; Norden and Blumenfeld, 2002; Blumenfeld et al., 2004; Englot et al., 2009, 2010. Previous studies have shown that the activation state of the frontoparietal region was associated with loss of consciousness (Untergehrer et al., 2014). CPS also had a distinct “spatial shift of slow waves”, that was, after a seizure, slow waves spread from the frontal cortex to the contralateral parietal and temporal lobes. However, this phenomenon was not found in SPS individuals (Yang et al., 2012). Similar results were seen in neuroimaging. Both SPECT and fMRI detected a reduction in subcortical cerebral blood oxygen level-dependent signals in the frontoparietal region in patients with generalized epilepsy (Gotman et al., 2005; Bai et al., 2010). Similarly, individuals with other unconscious states, such as coma, anesthesia, and brain death, showed impaired functional integration of the resembled cortex (Noirhomme et al., 2010; Crone et al., 2013; Gruenbaum, 2021). Therefore, we speculated that in TLE, the presence of abnormal functional connectivity in the frontoparietal cortex had led to impairment of consciousness. Aberrant neuronal discharge in **patients with** CPS activated significant network disturbances at a later stage, which in turn led to frontoparietal network abnormalities that clinically manifested as impaired consciousness.

### Possible causes of automatism in patients with CPS

Increased epilepsy network coherence was a pathophysiology of epilepsy semiotics (Chauvel and McGonigal, 2014). Semiology depended on the interaction of epileptogenic focus and dissemination targets (Maillard et al., 2004). Automatism was considered to be one of the most common symptoms of CPS. About 75% of individuals with CPS might present with buccal and tongue movements, including smacking, swallowing, and spitting, called oral automatisms (OAAs) (Maldonado et al., 1988; Janati et al., 1990; Kramer et al., 1997).

Automatisms had been proposed to be associated with widespread cortical excitation. Several previous stimulation studies had shown that stimulation of frontal, insular, and temporal cortex or amygdala regions could induce automatisms (Maestro et al., 2008). The mechanism by which OAAs arose was the synchronous propagation of brain waves in the temporal–insular–parietal lobes, disturbing the cortical masticatory region. The abnormal cerebral cortex triggered the emergence of oral movements (Aupy et al., 2018). Similarly, individuals with preserved verbal responsiveness had lower frequencies of perfusion in ipsilateral parietal regions during interictal episodes of automatisms (Park et al., 2018). In our study, patients in the CPS group exhibited more complex clinical symptoms in addition to impaired consciousness compared to patients with SPS. There was a prominent difference in the functional connectivity in the CPS group. This further demonstrates that the clinical behavioral differences in **patients with** CPS were likely due to alterations in network connectivity between the limbic system and parietal lobes during the late stages of neuronal firing.

### Limitations

The present study has several limitations. First, since the number of features was much more than the number of subjects used in classification, we used the F-score method to measure the classification ability of each input feature so as to select the most important edges for making accurate predictions (Guyon and Elissee, 2003). However, the F-score method did not take the mutual information between features into account (Chen et al., 2006), and it can only be used for two classifications. Once the number of classifications was more than two, the F-score method will fail. Second, an insufficient sample size of subjects was still a factor that cannot be ignored due to the limitation of the research. Due to the small sample size of individuals with simple clinical SPS or CPS, to strictly enroll the criteria, we only absorbed individuals whose timing of onset was short and the frequency of clinical seizure was low. Thus, there were only individuals who were up to standard with a single seizure form.

## Conclusions

In this research, we divided the individuals with TLE into two subtypes according to different clinical symptoms, SPS and CPS, and constructed an SVM classifier to classify the functional connectivity corresponding to these two types of individuals. Finally, a classification accuracy of 78.52% was achieved when we used the already existing SVM classifier on the testing set. To further verify the stability of the results above, we redesigned the data set allocation method and extracted the features, and finally obtained 81.37% classification accuracy. The final results showed that the nodes and edges involved in these features extracted by the above two methods had a high coincidence. Moreover, the two groups of individuals had significant differences in the connection between the parietal lobe and other brain regions. This finding may provide an idea for studying the pathogenesis of CPS or new thinking that can be used to research refractory epilepsy.

## Data availability statement

The original contributions presented in the study are included in the article/supplementary material, further inquiries can be directed to the corresponding author.

## Ethics statement

The studies involving human participants were reviewed and approved by Ethics Committee of Affiliated Hospital of Nanjing University of Traditional Chinese Medicine. The patients/participants provided their written informed consent to participate in this study.

## Author contributions

TW contributed to conceptualization, methodology, software, data curation, and writing. YW contributed to conceptualization, methodology, and supervision. ZL and YL contributed to methodology and software. YZ and YW contributed to writing the manuscript. YZ and XX contributed to conceptualization and supervision. All authors have read and agreed to the published version of the manuscript.

## Funding

This work was partially supported by the National Natural Science Foundation of China (Grant No. 82172022).

## Conflict of interest

The authors declare that the research was conducted in the absence of any commercial or financial relationships that could be construed as a potential conflict of interest.

## Publisher's note

All claims expressed in this article are solely those of the authors and do not necessarily represent those of their affiliated organizations, or those of the publisher, the editors and the reviewers. Any product that may be evaluated in this article, or claim that may be made by its manufacturer, is not guaranteed or endorsed by the publisher.
